# Is It Time for Ocrelizumab Extended Interval Dosing in Relapsing Remitting MS? Evidence from An Italian Multicenter Experience During the COVID-19 Pandemic

**DOI:** 10.1007/s13311-022-01289-6

**Published:** 2022-08-29

**Authors:** Aurora Zanghì, Carlo Avolio, Elisabetta Signoriello, Gianmarco Abbadessa, Maria Cellerino, Diana Ferraro, Christian Messina, Stefania Barone, Graziella Callari, Elena Tsantes, Patrizia Sola, Paola Valentino, Franco Granella, Francesco Patti, Giacomo Lus, Simona Bonavita, Matilde Inglese, Emanuele D’Amico

**Affiliations:** 1 UOC Neurology, Sant’Elia Hospital, Caltanissetta, Italy; 2 Department of Medical and Surgical Sciences, University of Foggia, Foggia, Italy; 3 Head of Multiple Sclerosis Center, Dept. of Neurosciences, Policlinico Riuniti Hospital, Foggia, Italy; 4 Multiple Sclerosis Center, II Division of Neurology, Department of Clinical and Experimental Medicine, Second University of Naples, Naples, Italy; 5 Dipartimento di Scienze Mediche e Chirurgiche Avanzate, Università della Campania Luigi Vanvitelli, Piazza Miraglia, 2, 80138 Naples, Italy; 6 Department of Neuroscience, Rehabilitation, Ophthalmology, Genetics, and Mother-Child Health (DINOGMI), University of Genoa, Genoa, Italy; 7 Department “G.F. Ingrassia”, MS Center University of Catania, Catania, Italy; 8 Azienda Ospedaliera Universitaria “Mater Domini”, Catanzaro, Italy; 9 Institute Foundation “G. Giglio”, Cefalù, Italy; 10 Department of General Medicine, Parma University Hospital, Parma, Italy; 11 University of Modena and Reggio Emilia, Modena, Italy; 12 Unit of Neurosciences, Department of Medicine and Surgery, University of Parma, Parma, Italy; 13 Multiple Sclerosis Centre, Department of General Medicine, Parma University Hospital, Parma, Italy; 14 IRCCS Ospedale Policlinico San Martino, Genoa, Italy

**Keywords:** Ocrelizumab, Extended interval dosing, Standard interval dosing, MRI, Disease activity

## Abstract

In the COVID-19 pandemic era, safety concerns have been raised regarding the risk of severe infection following administration of ocrelizumab (OCR), a B-cell-depleting therapy. We enrolled all relapsing remitting multiple sclerosis (RRMS) patients who received maintenance doses of OCR from January 2020 to June 2021. Data were extracted in December 2021. Standard interval dosing (SID) was defined as a regular maintenance interval of OCR infusion every 6 months, whereas extended interval dosing (EID) was defined as an OCR infusion delay of at least 4 weeks. Three infusions were considered in defining SID vs. EID (infusions A, B, and C). Infusion A was the last infusion before January 2020. The primary study outcome was a comparison of disease activity during the A-C interval, which was defined as either clinical (new relapses) or radiological (new lesions on T1-gadolinium or T2-weighted magnetic resonance imaging (MRI) sequences). Second, we aimed to assess confirmed disability progression (CDP). A total cohort of 278 patients (174 on SID and 104 on EID) was enrolled. Patients who received OCR on EID had a longer disease duration and a higher rate of vaccination against severe acute respiratory syndrome-coronavirus 2 (p < 0.05). EID was associated with an increased risk of MRI activity during the A-C interval (OR 5.373, 95% CI 1.203–24.001, p = 0.028). Being on SID or EID did not influence CDP (V-Cramer 0.47, p = 0.342). EID seemed to be associated with a higher risk of MRI activity in our cohort. EID needs to be carefully considered for OCR-treated patients.

**Supplementary Information:**

The online version contains supplementary material available at 10.1007/s13311-022-01289-6.

## Introduction

The COVID-19 pandemic has raised several concerns regarding the healthcare strategies for people with multiple sclerosis (MS), particularly in terms of the safety of specific disease-modifying therapies (DMTs), especially immunosuppressant ones [1–3]. During the peak of the COVID-19 pandemic, almost all in-person routine or elective neurological visits were stopped, and many scheduled appointments for infusion therapy were postponed. Such delays in treatment were reportedly not caused by fear of immunosuppressive drug use but rather by the general fear of contracting a fatal disease, which is often the case during traveling and hospital visits [4]. In the context of COVID-19, B-cell-depleting therapies may not only be accompanied by higher rates of infection but also influence the severity and mortality of such infections, although systematic data are lacking [2, 4–6]. In general, pulsed anti-CD20 therapies, such as ocrelizumab (OCR), were associated with an increased risk of hospitalization or intensive care admission in patients with COVID-19 [7]. The interval between infusions for OCR-treated patients is usually determined by selective immunosuppression of peripheral B cells, and standard interval dosing (SID) is scheduled every six months [8, 9]. Extended interval dosing (EID) for OCR has not been licensed or characterized, even if some real-world studies have investigated its efficacy and safety, and it is usually defined as an extension dose every 4 weeks or more [10]. However, systematic EID information for OCR-treated patients remains lacking. For these reasons, we decided to collect real-world Italian data on patients with relapsing remitting MS (RRMS) treated with OCR during the COVID-19 pandemic. We aimed to compare SID and EID regimens primarily in terms of disease activity and secondarily regarding confirmed disability progression (CDP).

## Methods

### Setting

A multicenter Italian real-world retrospective study was designed, in which data were collected from nine tertiary Italian MS centers.

Using iMed© software (iMed, Merck Serono SA—Geneva, Switzerland) as the data entry portal, the treating clinics used rigorous quality assurance procedures for the patient health records, certified according to the iMed© software data coordinator regulation [11]. Anonymized clinical data of MS patients were extracted on December 31, 2021.

### Participants

We included all RRMS patients who had an OCR prescription in accordance with treatment procedures and guidelines approved by the European and Italian Medicines Agencies [8, 9].

The inclusion criteria were as follows: 1) an RRMS diagnosis according to the revised McDonald criteria [12] and 2) previous completion of the first OCR initial treatment cycles (2 × 300 mg with a 2-week interval). Patients with primary progressive MS (PPMS) or without follow-up data were excluded.

### Procedures and Covariate Definitions

**Fig. 1:**
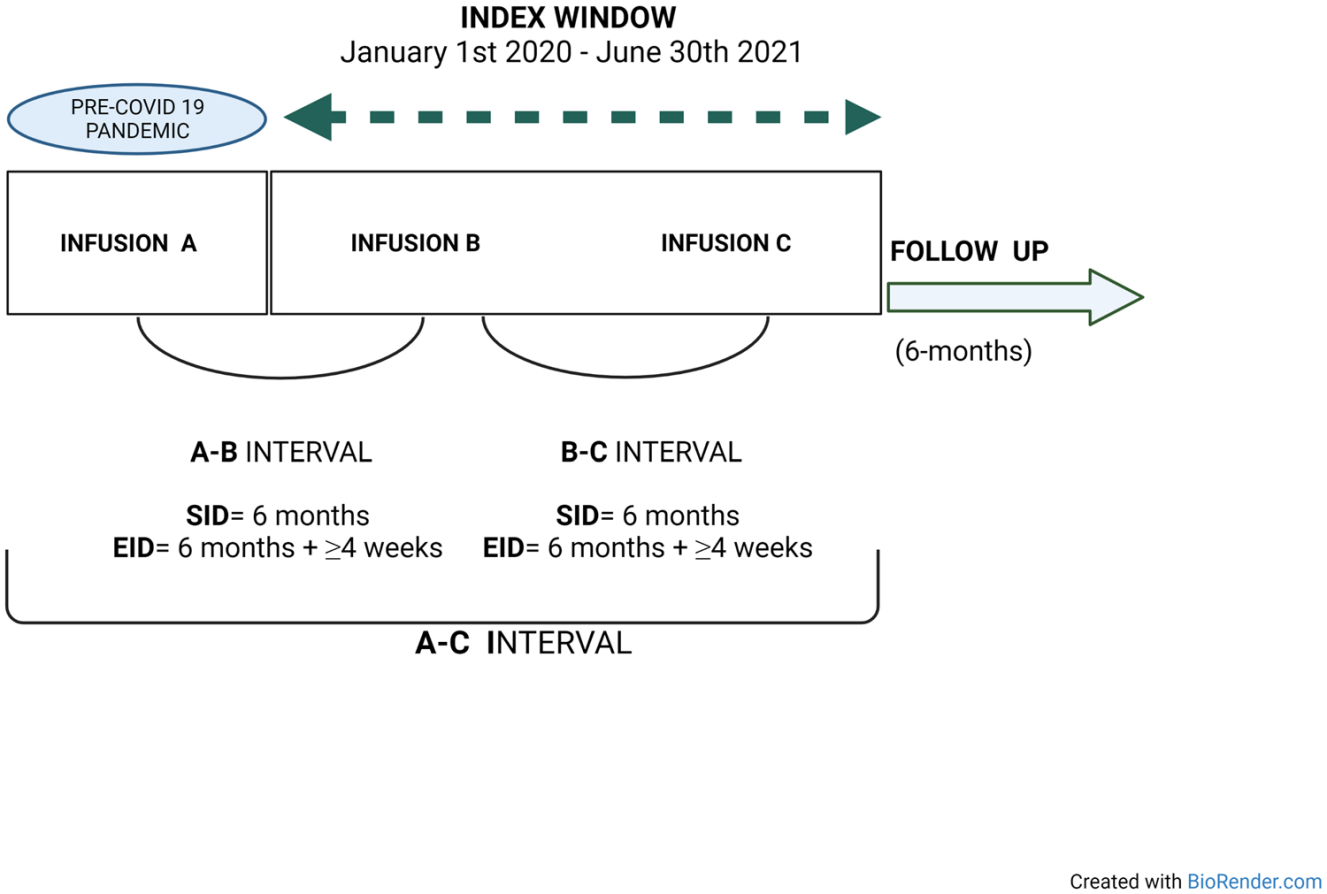
Flowchart of the study procedure. SID was defined as a regular maintenance interval of OCR infusions every 6 months, whereas EID was defined an OCR infusion delay of at least 4 weeks (6 months + ≥ 4 weeks delay). Infusion A was the last ocrelizumab infusion (second 300 mg cycle or 600 mg maintenance infusion) before January 2020, while infusions B and C (always 600 mg standard maintenance dose) were the subsequent infusions. Infusion C was the last OCR infusion. The observation period in which either SID or EID took place (always maintenance cycle, 600 mg) was from January 2020 to June 2021. We considered a single interval from infusions A to C, defined as the A-C interval. EID, extended interval dosing; SID, standard interval dosing

The observation period in which either SID or EID took place (always maintenance cycle, 600 mg) was from January 2020 to June 2021 (see Fig.1). SID was defined as a regular maintenance interval of OCR infusions every 6 months, whereas EID was defined an OCR infusion delay of at least 4 weeks (6 months + ≥ 4 weeks delay). Three infusions were considered in defining SID vs. EID, defined as follows: infusion A was the last OCR infusion (second 300 mg cycle or 600 mg maintenance infusion) before January 2020, while infusions B and C (always 600 mg standard maintenance dose) were the subsequent infusions, administered between January 2020 and June 2021; infusion C was the last OCR infusion. According to this definition, we considered a single interval from infusions A to C, defined as the A-C interval (see Fig. 1).

Disability was assessed with the Expanded Disability Status Scale (EDSS) by a Neurostatus-certified MS specialist [13]. Magnetic resonance imaging (MRI) data were acquired on 1.5-T scanners (the same at each center from baseline to the end of the follow-up) and included T2- and pre- and postcontrast T1-weighted sequences. Postcontrast T1-weighted sequences were acquired after intravenous injection of gadolinium contrast agent (0.1 mmol/kg). The numbers of brain MRI lesions on the T2-weighted, T1-weighted, and postcontrast T1-weighted sequences were recorded at every 6-month follow-up, as is standard in our clinical practice. A cerebral MRI acquired within three months before infusion A was considered the baseline MRI.

Clinical, MRI and disability outcomes were collected during the A-C interval and six months after infusion C (Fig. 1). We also collected peripheral blood CD19 + B-cell counts before each infusion, and the reasons for the use of either the SID or EID regimen were collected. Vaccination status against severe acute respiratory syndrome coronavirus 2 virus (SARS-CoV-2) was collected. In Italy, vaccination against SARS-CoV-2 started in January 2021 and was recommended to all people with chronic conditions from April 2021 [14].

### Study Outcomes

Our first aim was to investigate any association between EID and disease activity during the A-C interval. Disease activity was considered as two different outcomes: clinical and MRI activity.

Clinical activity was defined by the presence of relapses, which was defined as the occurrence of new symptom(s) or the exacerbation of existing symptom(s) persisting for at least 24 h in the absence of concurrent illness or fever, occurring at least 30 days after a previous relapse [15]. Definition of relapses among centers is standardized. MRI activity was defined as new or enlarged T2-weighted or T1-weighted gadolinium-enhanced lesions.

Second, we aimed to assess CDP with standardized neurologic examinations that are usually scheduled every six months.

CDP was considered relevant if 2 independent clinical assessments indicated an increase in the EDSS score as follows: 1.5 points (baseline EDSS 0.0), 1.0 point (baseline EDSS 1.0–5.5), and 0.5 points (baseline EDSS ≥ 5.5).

Ancillary, the patients were followed up six months after the last infusion through the evaluation of the proportion of patients with no evidence of disease activity, as defined by the three-parameter no evidence of disease activity (NEDA-3) status, a composite that consisted of (a) the absence of clinical relapses; (b) no CDP sustained for 12 weeks (as measured by the EDSS); (c) the absence of T1 gadolinium-enhanced (Gad +) brain lesions as well as the absence of any new/newly enlarging T2 brain lesions [16].

Peripheral blood CD19 + B-cell counts were collected before each infusion, and depletion was defined as < 10 cells/μL.

### Statistical Analysis

Data were described according to the nature of the variables. All patient characteristics are reported as the frequency (%) for categorical variables and the mean ± standard deviation (SD) or median with interquartile range (IQR) for continuous variables. A P value of 0.05 was considered statistically significant for all analyses. The Chi-squared test or Fisher’s exact test was applied when necessary to evaluate the association between categorical variables. The t test (one or two samples) or nonparametric Mann–Whitney U test, when appropriate, was applied according to the data distribution. For the one-sample test, the standardized mean difference and confidence interval were reported.

Logit regression models were built for the two outcomes “clinical activity” and “MRI activity” for the A-C interval.

In the first phase of the data analyses, a generalized linear mixed model with random intercepts was built using id center as the random effect [17]. Analysis of the covariance of the random intercepts of each model did not reach significance (“clinical activity” model, Z-Wald 1.548, p = 0.122, “MRI activity” model, Z-Wald 0.710, p = 0.478). Then, the logit model with fixed effects with the best statistical properties was chosen according to the Akaike information criterion [29]. For each model, the following variables were inserted: EID status (0 = SID, 1 = EID, dichotomic), age (continuous variable), sex (0 = male, 1 = female, dichotomic), number of DMTs before OCR (continuous variable), time on OCR from start to infusion A (continuous variable, months), relapses in the year before infusion A (0 = no, 1 = yes, dichotomic), MRI activity in the year before infusion A (0 = no, 1 = yes, dichotomic), EDSS score before infusion A (continuous), and CD19 + B-cell depletion rate before infusions A, B, and C (0 = depletion, 1 = no depletion, dichotomic).

Vaccination was not inserted into the model because no events occurred after the vaccination dates.

For dichotomous variables, the last value was considered the reference.

In the multivariable analysis, all variables with a p value < 0.10 in the univariate analysis were considered. The results are presented as an estimate of the odd ratio (OR) and the corresponding 95% confidence interval (CI).

R^2^ was reported as a measure of the percentage of total variation in the dependent variable that was accounted for by the independent variable. CDP was compared using the V-Cramer coefficient.

A bias-corrected and accelerated bootstrap sensitivity analysis (number of iterations = 1000) was conducted for both logit models [18].

The disease activity recorded six months after infusion C was reported as the NEDA-3 score and compared with contingency tables and the Chi-square test. All analyses were performed using SPSS V.21 statistical software.

## Ethics Statement

The Ethics Committee of the University of Foggia (Italy) approved the study (14/CE/2021). The study was conducted in accordance with the ethical principles of the Declaration of Helsinki.

## Results

### Participants

**Fig. 2:**
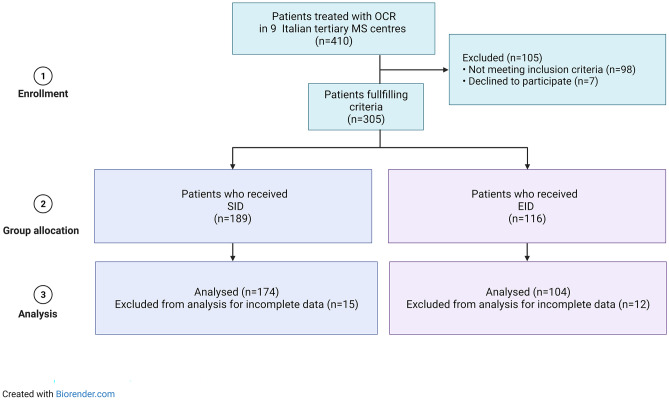
Flow chart of population enrollment. This flowchart depicts how the 278 ocrelizumab-treated patients with relapsing–remitting multiple sclerosis (RRMS) were identified. The source population was all patients with multiple sclerosis (MS) treated with ocrelizumab in 9 Italian centers during the period between January 2020 and June 2021. We excluded patients who did not meet the inclusion criteria, who denied their consent to participate or with incomplete data on electronical medical records. EID, extended interval dosing; SID, standard interval dosing; MS, multiple sclerosis; OCR, ocrelizumab

From a total cohort of 410 patients treated with OCR, 278 patients fulfilled the inclusion criteria and were included in the analyses (Fig. 2). A list of participating centers is available in Appendix e1.

All patients received two infusions (B and C) along the A-C interval; 174 received only SID infusions, while 104 received at least one EID infusion. Table 1 shows the baseline characteristics of patients on EID versus those of patients on SID. Patients on EID had a longer disease duration (12.1 ± 7.9 vs. 9.7 ± 7, respectively, p = 0.011) and a higher rate of vaccination against SARS-CoV-2 (67/104, 63.1% vs. 85/174, 48.6%, respectively, p = 0.025) than those on SID. The rate of B-cell depletion before the three infusions did not differ between the two groups (p > 0.05 for all). The most frequent reason for choosing an EID regimen was concern related to the risk of contracting COVID-19 in a hospital environment and the lack of certain information about the consequences of COVID-19 and anti-CD20 therapy (70/104, 67.3%).

**Table 1:** Whole cohort and SID/EID group characteristics

Variables**	**TOTAL** **(n = 278)**	**SID** **(n = 174)**	**EID** **(n = 104)**	p value*
**A-C interval duration (months)**	21 ± 12.2	10.3 ± 3.6	15.1 ± 2.5	** < 0.01**
**Age**	43.2 ± 11.3	42.9 ± 11.7	43.6 ± 10.5	0.623
**Female, n (%)**	178(64)	106 (60.9)	72(69.2)	0.204
**Disease duration (years)**	10.6 ± 7.5	9.7 ± 7	12.1 ± 7.9	**0.011**
**Naïve, n (%)**	51 (18.3)	38 (21.8)	13 (12.5)	0**.**073
**N. of DMTs before OCR**	1.9 ± 1.5	1.8 ± 2.2	2.1 ± 1.6	0.073
**EDSS score before infusion A, median [IQR] (all)**	3.0 [2.0–6.0]	3.0 [1.0–6.0]	3.0 [2.0–6.0]	0.411
**Patients who relapsed in the year before infusion A,** **n (%)**	17 (6.1)	11 (6.3)	6 (5.8)	0.942
**Patients with MRI activity in the year before infusion A, n (%)**	25 (8.9)	14 (8)	9 (8.7)	0.962
**Time on OCR (from start to infusion A, months)**	29.2 ± 10.8	29.1 ± 12.1	29.2 ± 8.1	0.964
**Vaccination against SARS-CoV-2, n (%)**	122 (43.8)	85 (48.9)	67 (64.4)	**0.025**
**Reasons for EID**				
*Concerns related to the risk of contracting COVID-19/lack of certain information about the consequences of COVID-19 and OCR therapy, n (%)*	-	-	70 (67.3)	
*Delay due to vaccination against SARS-CoV-2, n (%)*	-	-	34 (32.7)	
**CD19 + B-cell depletion before infusion A, n (%)**	139 (50)	88 (50.6)	51 (49)	0.901
**CD19 + B-cell depletion before infusion B, n (%)**	182 (65.5)	119 (68.4)	63 (60.6)	0.241
**CD19 + B-cell depletion before infusion C, n (%)**	179 (64.4)	117 (67.2)	62 (59.6)	0.197

*DMTs* disease modifying therapies, *EID* extended interval dosing, *EDSS* expanded disability status scale; MRI, magnetic resonance imaging; N. number, *OCR* ocrelizumab, *SARS-CoV-2* severe acute respiratory syndrome coronavirus 2

*via t test, Mann–Whitney U test or chi-squared test; **data are reported as the mean ± standard deviation unless otherwise specified. Statistically significant values are shown in bold

### A-C interval Analysis

During the A-C interval, 24 relapses occurred in 18 patients (3 patients with 2 relapses and one with 4 relapses); two of them received EID. Concomitant MRI activity was recorded in two patients.

**Fig. 3:**
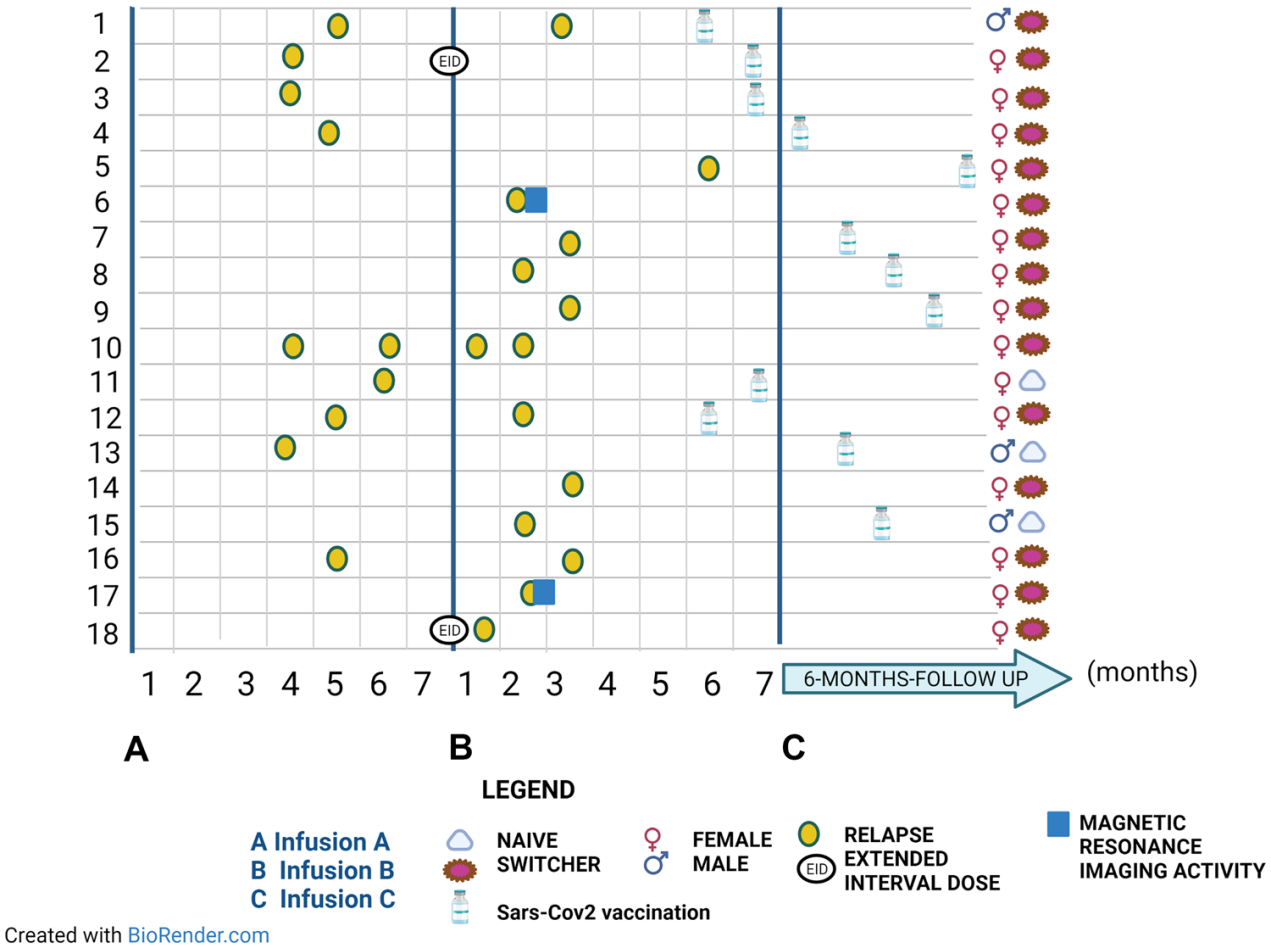
Relapse distribution during the A-C interval. Relapses are allocated along the timeline. The eventual extended interval regimen was specified; gender and naive/switcher status with respect to therapy were also entered for each patient. The eventual vaccination against Sars-Cov2 has been inserted along the timeline. Concomitant MRI activity was specified. Sars-Cov2, Severe acute respiratory syndrome-Coronavirus2

Figure 3 shows the relapse distribution over the investigated period.

Patients with relapses had a lower median time on OCR therapy than those without relapses (24 months, IQR 20–31 vs. 29 months, IQR 24–35, p = 0.026). No other significant differences were found between the two subgroups. The one-sample t test revealed that patients with relapses had a higher number of previous DMTs before OCR (standardized mean difference 2.7, standard error difference 0.3, 95% CI 1.8–3.1, p < 0.001) than the standardized population.

EID was not associated (OR 2.827, 95% CI 0.929–8.599) with the outcome of clinical disease activity. In the multivariable model were retained as predictors the CD 19 + B-cell depletion rate before infusion C (OR 0.750, 95% CI 0.032–0.948, p = 0.043) and the time on OCR therapy (OR 0.821, 95% CI 0.736–0.915, p = 0.001), both with an inverse relation (Table 2).

**Table 2:** Univariate and multivariable models for “clinical activity”

**Independent variable**	**Univariate analysis**		Multivariable analysis*	
	**OR (95% CI)**	**P value**	**OR (95% CI)**	**P value**
**Sex**	2.706 (0.888–8.24)	0.080		
**Age (years)**	0.976 (0.939–1.015)	0.231		
**Disease duration (years)**	0.979 (0.919–1.043)	0.513		
**N. of DMTs before OCR**	0.100 (0.958–1.642)	0.100		
**EDSS before infusion A**	1.138 (0.855–1.516)	0.374		
**Patients with relapses in the year before infusion A**	0.661 (0.188–2.322)	0.518		
**Patients with MRI activity in the year** **before infusion A**	1.773 (0.699–4.496)	0.228		
**Time on OCR therapy (from start to infusion A, months)**	0.930 (0.877–0.986)	0.016	0.821 (0.736–0.915)	0.001
**EID**	2.827 (0.929–8.599)	0.070		
**CD19 + B-cell depletion rate** **before infusion A**	1.428 (0.550–3.702)	0.464		
**CD19 + B-cell depletion rate** **before infusion B**	0.914 (0.306–2.735)	0.873		
**CD19 + B-cell depletion rate** **before infusion C**	0.164 (0.042–0.638)	0.009	0.750 (0.032–0.948)	0.043

*OR* odds ratio, *DMTs* disease modifying therapies, *EID* extended interval dosing, *EDSS* expanded disability status scale, *MRI* magnetic resonance imaging, *N* number, *OCR* ocrelizumab, *SARS-CoV-2* severe acute respiratory syndrome coronavirus 2

^*^R^2^ 0.5

**Fig. 4:**
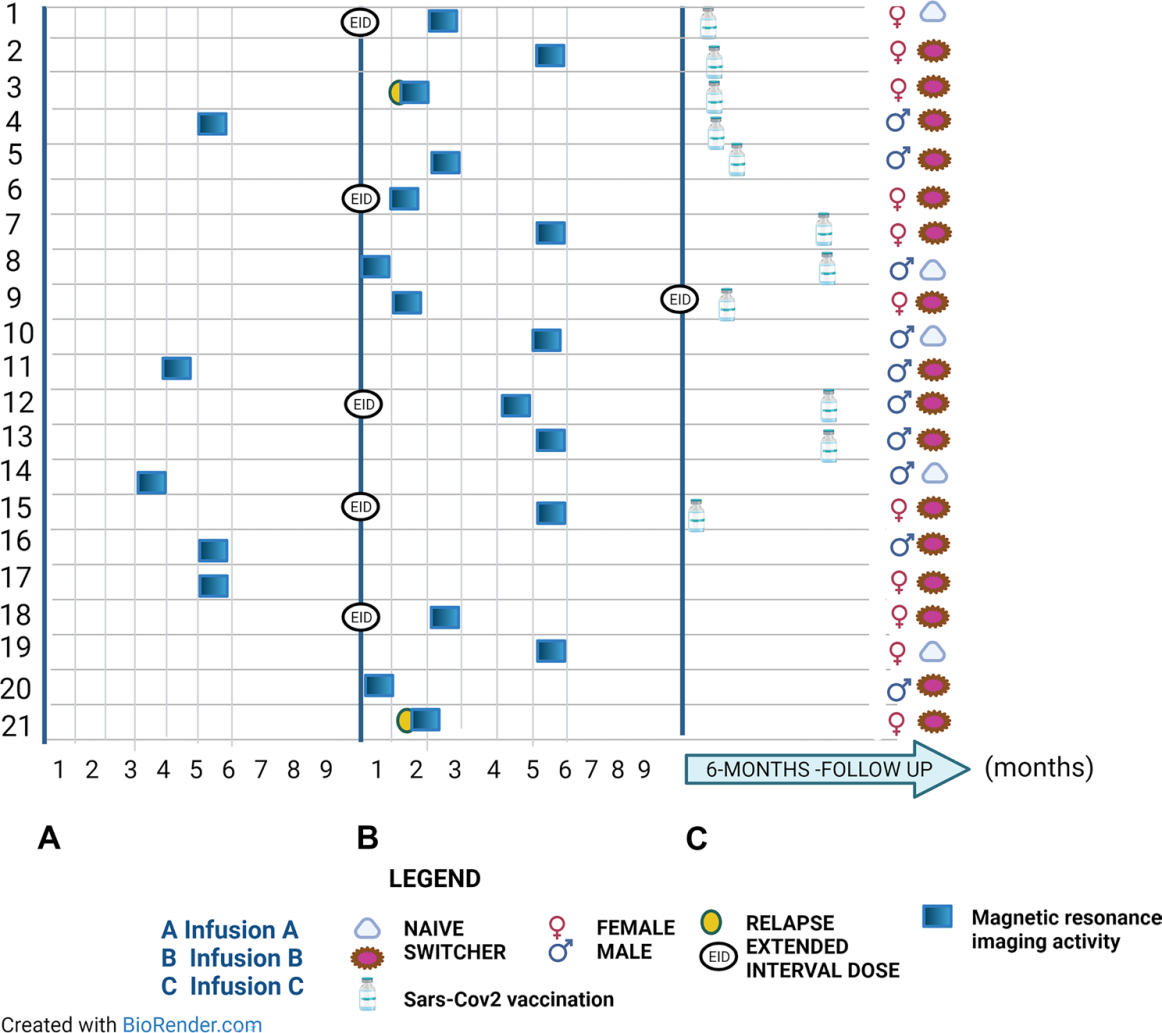
MRI activity distribution during the A-C interval. MRI activity was allocated along the timeline. The eventual extended interval regimen was specified; gender and naive/switcher status with respect to therapy were also entered for each patient. The eventual vaccination against Sars-Cov2 has been inserted along the timeline. Concomitant clinical activity was specified. Sars-Cov2, Severe acute respiratory syndrome-Coronavirus2

A total of 21 patients had MRI activity during the A-C interval, six of whom were on EID (Fig. 4). All patients had baseline MRI.

Patients with MRI activity had, on average, a lower disease duration than those without it (5 years, IQR 3–8 vs. 9 years, IQR 5–17, p = 0.003). No other significant differences were found between the two subgroups.

In the logit multivariable model for MRI activity (Table 3), EID was associated with the outcome (OR 5.373, 95% CI 1.203–24.001, p = 0.028). Furthermore, disease duration (OR 0.904, 95% CI 0.824–0.992, p = 0.034) and CD19 + B-cell depletion rate before infusion C (OR 0.197, 95% CI 0.046–0.846, p = 0.029) were retained in the model, both with an inverse relation.

**Table 3:** Univariate and multivariable models for “MRI activity”

**Independent variable**	**Univariate analysis**		Multivariable analysis*	
	**OR (95% CI)**	**P value**	**OR (95% CI)**	**P value**
**Sex**	1.560 (0.648–3.758)	0.321		
**Age (years)**	0.977 (0.940–1.016)	0.252		
**Disease duration (years)**	0.901 (0.828–0.980)	0.015	0.904 (0.824–0.992)	0.034
**N. of DMTs before OCR**	0.794 (0.575–1.098)	0.161		
**EDSS score before infusion A**	0.722 (0.525–0.992)	0.055		
**Patients with relapses in the year before infusion A**	1.499 (.427–5.269)	0.528		
**Patients with MRI activity in the year** **before infusion A**	1.427 (0.579–3.521)	0.440		
**Time on OCR therapy (from start to infusion A, months)**	1.018 (0.979–1.059)	0.370		
**EID**	3.32 (1.127–9.970)	0.030	5.373 (1.203–24.001)	0.028
**CD19 + B-cell depletion rate** **before infusion A**	1.428 (0.550–3.702)	0.464		
**CD19 + B-cell depletion rate** **before infusion B**	0.914 (0.306–2.735)	0.873		
**CD19 + B-cell depletion rate** **before infusion C**	0.338 (0.100–0.890)	0.045	0.197 (0.046–0.846)	0.029

*OR* odds ratio, *DMTs* disease modifying therapies, *EID* extended interval dosing, *EDSS* expanded disability status scale, *MRI* magnetic resonance imaging, *N* number, *OCR* ocrelizumab, *SARS-CoV-2* severe acute respiratory syndrome coronavirus 2

^*^R^2^ 0.6

Generally, being on SID or EID did not influence CDP (6/174 vs. 6/104, V-Cramer 0.47, p = 0.342).

### Bootstrapping Sensitivity Analysis

The bootstrapping analysis for the logit model “clinical disease activity” confirmed the inverse relation with the CD 19 + B-cell depletion rate before infusion C (OR 0.798, 95% CI 0.567–0.967, p = 0.017) and the time on OCR therapy (OR 0.795, 95% CI 0.362- 0.825, p = 0.001). The bootstrapping analysis for the logit model “MRI activity” confirmed the direct relation with EID (OR 2.921, 95% CI 1.136–8.796, p = 0.037) and inverse relation with disease duration (OR 0.877, 95% CI 0.258–0.961, p = 0.010). The CD19 + B-cell depletion rate before infusion C (OR 0.434, 95% CI 0.255–1.810, p = 0.072) was no longer held in the model.

### Six-Month NEDA-3 Status After Infusion C

Six months after infusion C, a total of three relapses were recorded (mean time 3.4 ± 0.2 months), all in patients who had received only SID infusions previously. MRI activity was observed in 5 patients (mean time 2.9 ± 0.5 months), three of whom had received at least one previous EID infusion. No CDP events were recorded.

The six-month NEDA-3 status after infusion C did not differ between the two groups (SID 5/174 vs. EID 3/104, 97.1% for both).

## Discussion

In our multicenter real-world Italian cohort, EID was associated with an increased risk of MRI activity during the infusion interval observed, although it did not influence the risk of relapse occurrence.

Additionally, a CD19 + B-cell depletion before infusion C and a shorter time on OCR therapy were related to a higher risk of disease activity, while a shorter disease duration was related to a higher risk of MRI activity.

However, during the first 6 months after the last infusion, no significant differences were observed between the two groups in terms of NEDA-3 status.

Our report is a novel study in MS real-world clinical practice during the COVID-19 pandemic because it suggests the possibility of increased MRI activity associated with the EID regimen in OCR-treated patients.

MRI activity was associated with a more specific pattern of patients with a recent MS onset. As we have learned in long-term disease studies, the early stages of the disease are associated with high levels of inflammatory activity, which over the years are mostly replaced with progression phenomena independent of disease activity and linked to immune aging [19, 20].

Moreover, MRI biomarkers are more sensitive to disease activity than clinical outcome measures, as revealed by the concomitant occurrence of clinical relapses in only two patients in our cohort. These biomarkers have been used to monitor the response to anti-inflammatory agents in patients with RRMS [21].

To date, few reports have described the use of EID for OCR in MS patients during the COVID-19 era, and to our knowledge, this is the first report suggesting caution in use of the EID regimen due to MRI activity; although this does not impact disability, as revealed by the lower rate of CDP observed in both groups, we cannot exclude a negative impact on disability accrual with longer follow-up.

Generally, based on previous reports, CD20 depleting agents have been delayed by 1–3 months during the COVID-19 pandemic, and no major rebound in disease activity has been reported [10, 22–24]. Specifically, a recent German report evaluated the clinical consequences of EID in RRMS patients treated with OCR during the COVID-19 pandemic, comparing patients on EID (defined as ≥ 4-week delay of dose interval) with a control group on SID during the same period (January to December 2020) [10]. Here, three hundred eighteen patients with RRMS were longitudinally evaluated in 5 German centers. Multivariate logistic regression showed no association between treatment regimen and NEDA-3 status three months after EID (OR 1.266, 95% CI 0.695–2.305, *p* = 0.441) [10]. However, disease activity was defined as a composite score and not a single measure (clinical, radiological), and a short observation period was reported.

Another recent Croatian study evaluated 33 MS patients (11/31 PPMS) on EID during the COVID-19 pandemic [25]. Here, the OCR dosing delay was an independent predictor of CD19 + B-cell repopulation. However, comparisons with patients on SID were not available, and no MRI activity was collected.

Furthermore, an Italian report on 83 RRMS patients whose infusion was scheduled between March and December 2020 reported the experience of 56 patients whose treatments were delayed based on MS severity and SARS-CoV-2 infection risk profile with strict CD19 + B-cell repopulation rate monitoring [24]. In that study, none of the patients presented with relapses or active disease on MRI; however, the choice of EID was reserved for nonactive patients [24].

These findings suggest that the choice of therapy must take into consideration each patient’s disease course.

Additionally, in our report, the rate of CD19 + B-cell depletion before infusion C was found to be a protective factor against disease activity, regardless of whether SID or EID was used. This finding was confirmed in sensitivity analysis only for clinical activity. These data need to be further implemented and verified in largest population. However, it is in accordance with other reports that have suggested that OCR reinfusions could be scheduled when the CD19 B-cell count was ≥ 10 cells/µL [23]. Van Lierop et al. conducted an observational study on 159 MS patients who received personalized OCR dosing incentivized by the COVID-19 pandemic [23]. Redosing was scheduled when the CD19 B-cell count was ≥ 10 cells/µL (starting 24 weeks after the previous dose, repeated every 4 weeks). Here, the median interval until redosing or the last B-cell count was 34 (IQR 30–38) weeks [23]. No clinical relapses were reported, a minority of patients showed EDSS progression, and only 1.9% of 107 patients with a follow-up MRI scan showed radiological disease activity.

Another predictor of clinical activity was time on OCR therapy, which was inversely related to the risk of relapse occurrence, despite the median 24 months on therapy. Undoubtedly, due to the small number of patients with relapse, we were unable to fully characterize this subgroup. After mean standardization, these patients demonstrated a high number of previous DMTs. Other reports on the efficacy of OCR in switchers from other DMTs have shown that exit strategies from natalizumab/alemtuzumab/rituximab reveal good disease stabilization within the first 6 months or during the first year of therapy, while other data have been reported on switching from first-line therapies [26–31]. Our results are in accordance with a recent Italian work on 153 MS patients treated with OCR that suggested that better outcomes were observed in treatment-naïve patients at the 2-year follow-up [32].

The main strength of the study is the use of a large multicenter Italian real-world cohort with a longer observation period than other previously reported data.

In such a delicate era for clinicians and MS patients, we have attempted to characterize the target population to whom EID could be assigned. Our results suggest that EID in OCR-treated patients should be considered with caution, especially in RRMS-specific populations, because it could be associated with increased MRI activity; undoubtedly, prospectively collected data with longer follow-up are needed to uncover any association between this risk and long-term changes in the disease course trajectory.

It may be important to determine whether the extension of a single infusion interval significantly affects disease progression over a longer period of time in future studies; noninferiority studies should investigate the long-term approach of continuous EID in terms of clinical outcomes and safety concerns, as have been observed for other intravenous pulsed therapies (e.g., natalizumab) and those based on B-cell repopulation monitoring (e.g., rituximab) [33, 34].

The main limitation of the study is related to its observational nature. Comparative effectiveness research is prone to different kinds of bias. Firstly, selection and indication biases of the target patients to whom this approach should be reserved, which limited the robustness of the data on disease activity and combined with the relatively short observation period, prevented us from deriving a definitive conclusion about the possibility of EID in OCR-treated patients. Second, possible confounding bias that is the most-often cited source of potential bias. Consequently, each phenomenon had distinct consequences: confounding bias could compromise internal validity while selection/indication bias could compromise external validity.

Additionally, the reasons for choosing the EID regimen were too general, and undoubtedly more details on each patient would have added more information. During the COVID-19 era, as shown in recent Italian statistics, 1 in 2 centers demonstrated reduced performance, and 87 percent of centers had reduced neurological visits, exposing problems related to chronic patient management [35]. Italy was the first country in the Western world to have been heavily affected by COVID-19; its government and community, at all levels, reacted with great strength, reversing the trajectory of the epidemic with a series of science-based measures [36, 37]. Therefore, Italian MS patients could have also followed different paths with respect to patients from countries impacted by the pandemic in different ways or at later stages, when clinicians already had more definitive answers on infection and impact in immunomodulated/immunosuppressed patients.

The COVID-19 pandemic has placed new strains on our health and social systems. However, it has provided an opportunity and political momentum to invest in and reorganize these systems. We must use this moment to reconfirm and reenergize the MS community’s efforts and drive policymakers and decision-makers to take evidence-based action and ensure a holistic approach to care for all people with MS.

Our clinical practice-based study could aid clinicians in managing MS therapy during the COVID-19 pandemic and serve as a stimulus for the creation of systematic prospective EID studies in OCR-treated patients.

### Electronic supplementary material

Below is the link to the electronic supplementary material.

Supplementary file1 (PDF 1900 KB)

Supplementary file2 (PDF 1900 KB)

Supplementary file3 (PDF 1900 KB)

Supplementary file4 (PDF 1900 KB)

Supplementary file5 (PDF 1900 KB)

Supplementary file6 (PDF 1900 KB)

Supplementary file7 (PDF 1900 KB)

Supplementary file8 (PDF 1900 KB)

Supplementary file9 (PDF 1900 KB)

Supplementary file10 (PDF 1900 KB)

Supplementary file11 (PDF 1900 KB)

Supplementary file12 (PDF 1900 KB)

Supplementary file13 (PDF 1900 KB)

Supplementary file14 (PDF 1900 KB)

Supplementary file15 (PDF 1900 KB)

Supplementary file16 (PDF 1900 KB)

Supplementary file17 (PDF 1900 KB)

Supplementary file18 (PDF 1900 KB)

## Appendix e1 Participating Centers

1. Multiple Sclerosis Center, Department “G.F. Ingrassia”; MS center University of Catania, Catania, Italy
2. Multiple Sclerosis Center, Department of Medical and Surgical Sciences, University of Foggia, Foggia, Italy
3. Multiple Sclerosis Center, II Division of Neurology, Department of Clinical and Experimental Medicine, Second University of Naples, Naples, Italy
4. Multiple Sclerosis Center, Department of Advanced Medical and Surgical Sciences, University of Campania Luigi Vanvitelli, Naples, Italy
5. Multiple Sclerosis Center, Department of Neuroscience, Rehabilitation, Ophthalmology, Genetics, and Mother–Child Health (DINOGMI), University of Genoa, Genoa, Italy
6. Multiple Sclerosis Center, MD, University of Modena and Reggio Emilia, Modena, Italy
7. Multiple Sclerosis Center, University Hospital “Mater Domini”, Catanzaro, Italy
8. Multiple Sclerosis Center, Institute Foundation “G. Giglio”, Cefalù, Italy
9. Multiple Sclerosis Center, Department of General Medicine, Parma University Hospital, Parma, Italy

## Required Author Form

Disclosure forms provided by the author are available with the online version of this article.

## Protocol Approvals Standard, Registrations, and Patient Consents

The study protocol was approved by the local ethics committee (14/CE/2021) of the coordinating center (AOU Ospedali Riuniti di Foggia) and patients provided written informed consent. The study was conducted in accordance with the ethical principles of the Declaration of Helsinki and with the appropriate national regulations.
